# Radiologic-Histopathologic Correlation of Cerebral Microbleeds Using Pre-Mortem and Post-Mortem MRI

**DOI:** 10.1371/journal.pone.0167743

**Published:** 2016-12-09

**Authors:** Sven Haller, Marie-Louise Montandon, François Lazeyras, Max Scheffler, Stephan Meckel, Francois R. Herrmann, Panteleimon Giannakopoulos, Enikö Kövari

**Affiliations:** 1 Affidea Centre de Diagnostic Radiologique de Carouge CDRC, Carouge, Switzerland; 2 Medical School of the University of Geneva, Geneva, Switzerland; 3 Department of Surgical Sciences, Radiology, Uppsala University, Uppsala, Sweden; 4 Department of Neuroradiology, University Hospital Freiburg, Freiburg im Breisgau, Germany; 5 Department of Mental Health and Psychiatry, University Hospitals of Geneva, Geneva, Switzerland; 6 Department of Radiology and Medical Informatics, University of Geneva, Geneva, Switzerland; 7 Division of Geriatrics, Department of internal medicine, rehabilitation and geriatrics, Geneva University Hospitals, Geneva, Switzerland; 8 Medical Direction, University Hospitals of Geneva, Geneva, Switzerland; Henry Ford Health System, UNITED STATES

## Abstract

**Introduction:**

Cerebral microbleeds (CMB), also known as cerebral microhemorrhages, are small areas of susceptibility on brain magnetic resonance imaging (MRI), that are increasingly detected due to the higher availability of high-field MRI systems and dedicated pulse sequences. The prevalence of CMBs increases in cases with cognitive decline. The current investigation assessed the poorly investigated radiologic–histopathologic correlation of CMBs on MRI.

**Methods:**

The local ethical committee approved the current investigation. We retrospectively assessed a consecutive series of 1303 autopsy cases hospitalized in Geneva University Hospitals between 2000–2014. Of 112 cases with pre-mortem T2* sequences, we included 25 cases (mean age 77.3 ± 9.6, 9 females) with at least one CMB. We compared pre-mortem CMBs with targeted histopathology and post-mortem MRI.

**Results:**

25 cases had 31 CMB lesions detected by pre-mortem MRI. 25 additional CMB were detected on histopathology. 4 CMBs on pre-mortem MRI were false positives, resulting in a total of 52 CMBs. 27 CMBs on pre-mortem MRI were confirmed on histopathology, corresponding to a sensitivity or true positive rate of 51.9% (95% CI 37.6–66.0%). The false negative rate of pre-mortem MRI was 48.1% (95% CI 34.0–62.4%). Post-mortem MRI showed only 3 cases with additional CMBs. Overall, pre-mortem MRI significantly underestimated CMBs (p = 0.0001).

**Conclusions:**

Routine clinical brain MRI underestimates the prevalence of CMBs by approximately 50%, and 12% of radiologic pre-mortem MRI CMBs were false positives. Post-mortem MRI confirmed that this discordance is not explained by microbleeds occurring after the pre-mortem MRI.

## Introduction

Cerebral microbleeds (CMB), also referred to as cerebral microhemorrhages (CMH), are small hypointense lesions detected by susceptibly weighted brain magnetic resonance imaging (MRI) with variably cut-off size ranging from 5 to 10 mm [1–4]. The detection rate of CMB on MRI increases with increasing field strength, e.g. from 1.5T to 3T, but also with newer MR pulse sequences such as susceptibility weighted imaging (SWI) [5].

As discussed previously [6], the prevalence of CMB increases in cases with cognitive decline. The prevalence of CMB is higher in vascular dementia (VaD) (65% T2*, 86% SWI) compared to mild cognitive impairment (MCI; 20% T2*, 41% SWI) or Alzheimer’s disease (AD; 18% T2*, 48% SWI) [3, 7]. Newer susceptibility weighted imaging sequences such as SWI increase the detection of CMBs in MCI cases when compared to T2* imaging (23% in T2* versus 40% in SWI) [2]. However, this increase in detection rate does not improve the correlation with vascular risk factors or radiological markers of small-vessel disease.

The clinical repercussions of radiological CMBs are still a matter of debate [8–10]. An increased number of CMBs seems to increase the risk of stroke in elderly cohorts [11]. The number of CMBs is higher in cognitive decline / MCI relative to controls. However, it remains controversial whether increasing CMB load may predict further cognitive decline. For example, one study found that while the average number of CMBs at baseline did not differ between controls and MCI (11% and 14% respectively) more than three CMBs were present only within the progressive MCI group [8]. Another study reported a higher occurrence of microbleeds in progressive MCI (8 / 26 cases) as compared to stable MCI (1 / 23 cases) [9]. Two large-scale epidemiological studies report T2* CMB prevalence lower than 20% (11% in the Reykjavik AGES Study of 1962 participants, 18.7% in the Rotterdam study of 4759 participants) [11]. In contrast, histopathologic studies found CMB prevalence of 60–70% in very old individuals [12–14]. Taking into account the better detection of CMB by SWI as well as the inclusion of oldest cases in histopathologic studies, the difference in CMB prevalence between MRI scans and histopathology is still significant. It may reflect an insufficient detection of CMBs by clinical MRI. Alternatively, a high number of CMBs may occur during the last days of life after the last performed pre-mortem MRI.

To address this issue, we examined the concordance between radiological CMB on pre-mortem MRI with targeted histopathology in 25 consecutive autopsy cases containing a total number of 31 CMB on pre-mortem imaging (see Table 1). To explore the occurrence of CMB after the pre-mortem MRI and prior to death, post-mortem MRI scans were analysed in 20 of 25 cases.

**Table 1:** Distribution of cerebral microbleeds.

	Histology		
Pre-mortem MRI	+	-	Total
+	27	4	31
-	25	0	25
**Total**	52	4	56

Summary of the cerebral microbleeds observed on pre-mortem MRI and histopathology

## Materials and Methods

### Study population

**Fig 1:**
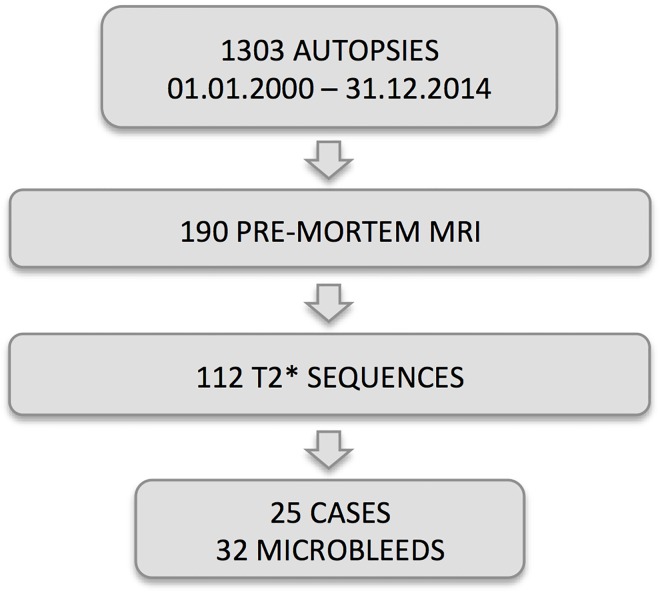
Schematic illustration of the study population in the current investigation.

The study was approved by the Ethics Committee of the University of Geneva, Switzerland. We retrospectively analyzed all of the patients with brain autopsy from the Geneva University Hospitals between 01.01.2000 and 31.12.2014. Among the 1303 available cases, 190 had pre-mortem MRI, and 112 had a T2* sequence. We analyzed only cases that had at least one small hypointense area on susceptibility weighted images compatible with CBM without adjacent substantial intracerebral pathology such as neoplasm and hemorrhage, which could interfere with the interpretation of the CBMs. We included 25 cases with a total of 31 CBM lesions (mean age 77.3 ± 9.6, 9 females) (see Fig 1). The interval between pre-mortem MRI and death was ranged from 0 to 76 months (mean 18 ± 24). Five cases had dementia (4 cases had AD– 2 of them were associated with vascular lesions, one with LBD; and one case of LBD with vascular lesions).

### Pre-mortem MRI

The pre-mortem MRI was performed during clinical workup over a period of 15 years. Consequently, MR machines and clinical protocols were variable and evolved over time. The minimal requirements for inclusion were an axial T2* image without relevant motion artifacts, and additional T1w and T2w and/or FLAIR sequences. Most cases, in particular cases with pathological findings, had also DWI and contrast-enhanced T1w images. The 25 cases included were scanned as follows: all cases had T2*, 8 cases SIEMENS 1.5 T, slice thickness 4 mm, TE = 25–26 ms; 9 cases SIEMENS 3T, slice thickness 3–5 mm,TE 16–20 ms; 5 cases PHILIPS 1.5T, slice thickness 3mm, TE 16ms; 3 cases PHILIPS 3T, slice thickness 4-5mm, TE 23ms.

Pre-mortem MRI was performed for different reasons, and only cases with normal brain findings were included: evaluation of cerebral status in extra-cerebral oncological cases (N = 7), search for ischemic brain lesions (N = 6), search for hemorrhage in head trauma (N = 4), extrapyramidal syndrome (N = 4), dementia (N = 2), dizziness (N = 1), sepsis (N = 1).

### Histopathology

For neuropathological routine analysis brains were cut earlier into 1-cm-thick coronal slabs (E.K. 20 years of experience). For the present study the location of each CMB was defined on the pre-mortem MRI and the corresponding CMB-containing slabs were used for further investigation. On these brain slices, post mortem MRI was performed (see section post-mortem MRI below). Thereafter, brain slices were embedded in paraffin and five 12-m(micro)m-thick serial sections (50 sections) were cut and every 10^th^ slide were stained with hematoxylin-eosin (HE) staining. On histological slides, CMBs were considered as focal accumulation of hemosiderin-containing macrophages (siderophages) around blood vessels [15] and corresponding to small perivascular bleeding, being at least several days of age.

### Post-mortem MRI

The selected coronal slices of approximately 1 cm thickness (see section histopathology above) were transferred into a waterbed. After careful removal of air bubbles using a Fisher Brand vacuum pump (Bel Art Product), imaging was performed on a 3T clinical MR scanner (Siemens PRISMA, Siemens Erlangen, Germany). We performed a 3D SWI sequence with the following key parameters; TE 20 ms, TR 43 ms, voxel size 0.7 x 0.7 x 0.7 mm3.

### Image analysis

CMBs were visually analyzed by one experienced neuroradiologist (S.H. with 16 years of experience). Each pre-mortem CMB was defined as true positive (TP), false positive (FP), or false negative (FN) as follows: A pre-mortem CMB was described as TP if it was also detected on histopathologic sections. It was described as FP, if it did not correspond to a microbleed on histopathologic examinations. It was considered as FN if it was identified on histopathology without a corresponding lesion on pre-mortem MRI. We have not measured the *true negative* (*TN*) rate (proportion of cases without radiological CBMs in pre-mortem MRI and absence of CMBs in histopathology) since this would require a detailed histopathologic analysis of the entire brain in thin slices, which is clearly beyond the scope of our work.

### Statistical analysis

The statistical unit is the number of CMB lesions detected, not the patients.

False positive and false negative rates were compared using Mc Nemar chi-square, enabling the statistical assessment of under or over-estimation of the CBM’s detection by pre-mortem MRI (F.R.H. 20 years of experience).

Statistics were computed with Stata release 14.1 with the exception of 95% confidence interval for 2 by 2 table which were obtained using DAGstats [16].

## Results

25 cases had 31 CMB lesions detected by pre-mortem MRI. 25 additional CMB were detected on histopathology. 4 CMBs on pre-mortem MRI were false positives (see below), resulting in a total of 52 confirmed CMBs

### True positive CMBs

**Fig 2:**
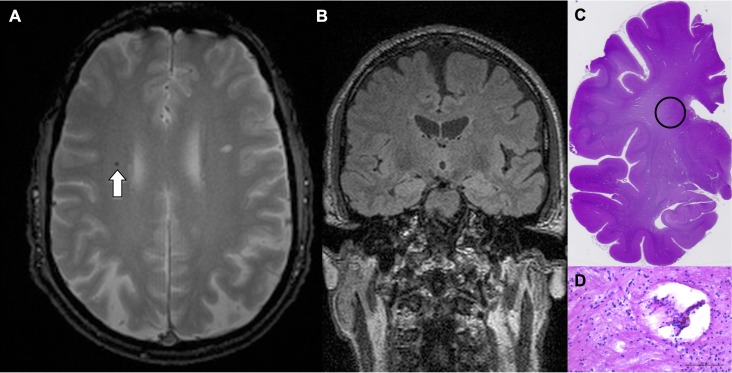
Fig 2 illustrates an example of a true-positive CMB in a 68 years-old man (at the time of pre-mortem MRI). Axial T2* illustrates one CMB in the frontal white matter (A). The corresponding coronal FLAIR (B) was used to guide targeted histopathology. Histological slide (C, haematoxylin-eosin-staining) illustrates the region of interest, and (D, haematoxylin-eosin-staining) corresponds to the encircled region on C with CMB.

27 CMBs on pre-mortem MRI were confirmed on histopathology, corresponding to a sensitivity or true positive rate of 27 / 52 or 51.9% (95% CI 37.6–66.0%). An example case is illustrated in Fig 2. Interestingly, all of the true positive CMBs were also detected in post-mortem MRI (when available).

### False positive CMBs

**Fig 3:**
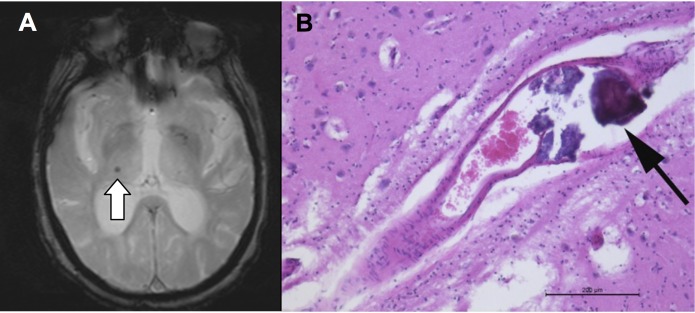
Fig 3 illustrates a false positive case. The hypo intense lesion on axial T2* image (A) in a 71 years-old woman at time of pre-mortem MRI corresponds to a calcification of an intrathalamic vessel (B, haematoxylin-eosin-staining) as false-positive CMB.

False positive (FP) CMBs are lesions visible on the pre-mortem MRI that did not correspond to a CMB on histopathology. We observed 4 FP. The real FP rate cannot be estimated due to the non-assessment of true negative TN. The FP lesions were two micro-calcifications, one periventricular hemorrhage, and one had no corresponding lesion on histopathology. An example case is illustrated in Fig 3.

### False negative CMBs

**Fig 4:**
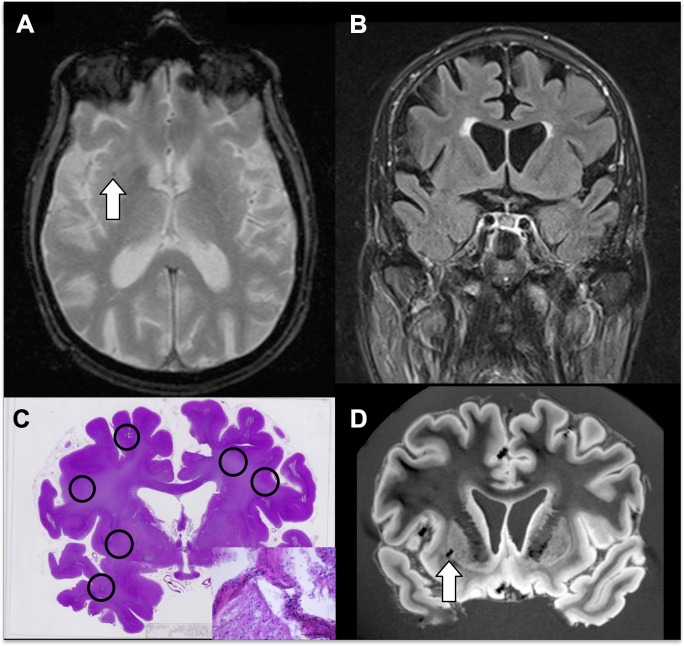
Example case illustrating one TP and 5 FN CMBs in an 82 years-old male at pre-mortem MRI. Axial T2* (A) demonstrates one right deep CMB. Coronal FLAIR (B) to target histopathology. Coronal histopathology (C, haematoxylin-eosin-staining) confirms the CMB on pre-mortem MRI, which is again present on post-mortem MRI SWI (D). Histopathology demonstrates 5 additional CMBs not evident on pre-mortem or post-mortem MRI. Insert shows haemosiderin-containing macrophages (arrows).

False negative (FN) CMBs are microbleeds identified on histopathology, without a corresponding lesion on pre-mortem MRI. Histopathology identified a total of 25 additional CMBs not visible on pre-mortem MRI, which corresponds to a FN rate of 48.1% (25/52; 95% CI 34.0–62.4%) of cases when considering only the pre-mortem MRI. To assess the possibility that CMBs might have occurred after the pre-mortem MRI, we additionally performed post-mortem MRI 20 / 25 cases. Post-mortem MRI showed additional CMBs as compared to pre-mortem MRI in only 3 cases. Of note, the histopathologic analysis was performed only in selected slices of the brain that corresponded to the location of the CMB on pre-mortem MRI. The additionally found CMBs on histopathology are therefore found in the limited area of the assessed brain, suggesting that the true FN rate might be even higher. An example case is illustrated in Fig 4.

The proportion of false positive cases of 4/56 = 7.1% is significantly different from the proportion of false negative cases of 25/56 = 44.5%, Mc Nemar chi-square at 15.21, with a P = 0.0001. This confirms the under-detection of CMB lesions by MRI (see Table 1).

## Discussion

The current investigation assessed the radiologic-histopathologic correlation of CMBs by the comparison of pre-mortem MRI, histopathology and post-mortem MRI. Overall, there was a correspondence between pre-mortem MRI and histopathology in 27 microbleeds, which corresponds to a TP rate or sensitivity of 51.9%. This correspondence is slightly lower than that reported previously in elderly cohorts [15, 17] pointing to the utility of MRI scans to identify in vivo histological CMBs in various clinical settings. However, we also found 4 false positive radiologic “CMB” on pre-mortem MRI that did not correspond to a microbleed on histopathology. In particular, we found two cases of micro calcification as “microbleed mimics”, one case of multiple fresh periventricular hemorrhages and one case without a lesion on histopathology. Consistent with these data, the few available radiological–histopathologic correlation studies of CMBs also demonstrate several cases with FP radiologic CMBs [15, 17]. This seems to be more frequent in pathological conditions. For instance, in a recent study assessing CMBs based on SWI in 10 patients with cerebral amyloid angiopathy, and a total of 38 lesions, the TP rate was of 48% (16/38) with 24% of FP cases including 7 small cavities, 1 dissection in the wall of a grossly distended vessel and 1 micro aneurysm [18]. False-positive CMB “mimics” include micro-dissection, micro aneurysm, micro calcifications and arteriolar pseudo calcification [15, 17, 18]. These two studies as well as three early studies in patients with Moyamoya [19], B cell lymphoma, pneumonia and subarachnoid hemorrhage [20] and ischemic stroke and gastric cancer [17] were all considered in a review article by Shoamanesh et al. [21]. These authors reported a total of 85 CMBs in 18 patients. Overall, 13 cases had no associated specific pathology, one case was due to vascular pseudo calcification, one case was a micro aneurysm and one case was a distended dissected vessel. A FP rate of 19% was retained.

Most importantly, pre-mortem MRI underestimated the CMBs with respect to histopathology in 48.1% of CMB lesions (based on pre-mortem MRI). To exclude the possibility that CMBs occurred in the delay between pre-mortem MRI and histopathology, we additionally performed post-mortem MRI in 20 / 25 cases. Interestingly, only 3 additional microbleeds were detected on post-mortem MRI. This means that in the majority of cases, microbleeds did not occur after the pre-mortem MRI and were truly under-estimated by the clinical pre-mortem MRI with respect to histopathology. Even when taking into account the post-mortem MRI and when calculating the false negative rate per case instead of per CMB, the FN rate remains at 24% (based on post-mortem MRI). Moreover, we would like to emphasize that the histopathologic analysis was performed only on selected brain slices targeted by the pre-mortem MRI and not on the entire brain. As a consequence, the real FN rate is therefore likely even higher, as we can assume that additional CMBs might be present in the remaining brain areas. One T2* study found a radiologic–histopathologic correlation in 21 of 34 microbleeds, which corresponds to a TP rate of 62%. Two out of 11 brains demonstrated hemosiderin deposits on histopathology that were not noted on MR imaging, which corresponds to a FN rate of 18% [15]. We want to emphasize that the primary aim of the additionally performed post-mortem MRI in the current investigation was to investigate whether micro-bleeds occurred in the interval between pre-mortem MRI and autopsy—as a potential explanation for the observed low sensitivity of pre-mortem MRI. The post-mortem MRI was therefore performed with similar parameters as standard pre-mortem imaging, evidently with the limitation of modifications of the imaging contrast in post-mortem MRI. Alternatively it would have been possible to perform very long data acquisitions of post-mortem MRI to improve the sensitivity of post-mortem MRI. As mentioned above, the focus of this investigation was not to obtain the best possible imaging performance of post-mortem MRI but to have comparable imaging performance of post-mortem MRI and pre-mortem MRI.

Few studies have assessed the radiologic-histopathologic correlation of CMBs at ultra high field at 7T. The large study by De Reuck et al. assessed postmortem brain sections from 20 Alzheimer patients including 79 hemorrhages of 1–3 mm in diameter on gross pathology and 163 hemorrhages invisible to the naked eye (200–500 um) [22]. The sensitivity to detect CMBs at ultra high field was excellent at 100%. Additionally detected FP lesions corresponded to perforating vessels filled with postmortem thrombi or iron / calcium deposits around vessels or in astrocytes. Since then, this group has published a series of 7T studies in various diseases including AD, frontotemporal lobar degeneration, LBD, amyotrophic lateral sclerosis, vascular dementia, progressive supranuclear palsy cerebral and amyloid angiopathy [23–30]. Another related issue concerns cortical microinfarcts well described by pathologists but to date under-diagnosed using clinical MR imaging. A recent study at 7T assessed such cortical microinfarcts [31]. Some of these cortical microinfarcts have a hemorrhagic component. The reported images suggest that the radiologic-histopathologic correlation of such hemorrhagic components was excellent, but sensitivity or specificity are not reported. Taken together, these findings imply that the radiologic-histopathologic correlation improves at 7T, yet this ultra-high scanners are not yet widely in use in clinical practice.

### Strengths and limitations

Major strengths of the current investigation with respect to previous related studies include the comparably large sample size collected over a period of 15 years. Moreover, in addition the current study performed post-mortem MRI to confirm that the additionally observed CMB on histopathology did not occur in the interval between pre-mortem MRI and death. Major weaknesses include the retrospective nature of the study and the fact that the pre-mortem MRI protocol varied over the years due to clinical practice and technical evolution. Finally, there is no gold standard for the true number of microbleeds on histology, as this would require a thin-slice analysis of the entire brain of all cases included in this study.

## Conclusions

Overall, routine clinical brain MRI significantly underestimates the prevalence of CMBs, and not all radiologic CMB lesions on pre-mortem MRI actually correspond to true microbleeds on histopathology.

## Abbreviations

AD: Alzheimer Dementia
CMB: cerebral microbleeds
CMH: cerebral microhaemorrhages
FN: false negative
FP: false positive
LBD: Lewy body dementia
MCI: mild cognitive impairment
MRI: magnetic resonance imaging
SWI: susceptibility weighted imaging
T2*: gradient-echo T2 weighted imaging
TN: true negative
TP: true positive
VaD: vascular dementia
